# Ultimate Shear Force of an Any Anchor Group Post-Installed into Concrete

**DOI:** 10.3390/ma16072608

**Published:** 2023-03-24

**Authors:** Paolo Foraboschi

**Affiliations:** Dipartimento Culture del Progetto, Università IUAV di Venezia, Dorsoduro 2206, 30123 Venice, Italy; paofor@iuav.it

**Keywords:** adhesive anchors, closed spaced anchors, hardened concrete anchors, mechanical expansion anchors, metal injection anchors, shear anchors, straight shaft anchors

## Abstract

This paper is devoted to the fastening system that consists of a number of anchors of approximately equal effective embedment depth, called “anchor group”, embedded into hardened concrete, used to transmit forces transverse to the anchors from an attachment to the concrete. The anchor group is far from the edges and is subjected to no more than marginal axial forces. Being post-installed, rather than cast-in, the embedded end of each anchor is not hooked, and no nuts, washers, or plates are attached to the embedded shaft. The paper focuses on the transverse forces that can be transmitted across an anchor group from an attachment to the concrete. The paper provides an analytical model for predicting the maximum (ultimate) shear force that an anchor group can bear, thus called “shear strength”. The model hence allows the structural designer to predict the shear strength of an anchor group post-installed into concrete. The model is based on five mechanical assumptions, which were established from a wide-ranging numerical analysis. Model predictions turned out to be, on average, 20% lower than the results of experiments performed on cast-in anchor groups borrowed from literature. The comparison verifies model accuracy, considering that the tested anchor groups benefitted from the extra-strength furnished by nuts and washers attached to the embedded shaft. Model predictions were also compared to code provisions; the former resulted to be up to one third of the latter. The paper presents and comments those comparisons, as well as all mathematical development. Applications of the model to wide-ranging case studies is presented and discussed as well.

## 1. Introduction: State-of-the-Art about Post-Installed Concrete Anchors

There has been much valuable work conducted in the field of adhesive and mechanical anchors intended for use in concrete, and concrete anchors have been the subject matter of many valuable papers. However, a detailed insight into those documents reveals that most of the papers are devoted to the anchor’s extraction strength, i.e., pull-out resistance [1,2,3,4,5,6,7,8], and to cast-in anchors, i.e., anchors installed in new structures, before pouring the concrete [9,10,11,12,13,14,15,16,17,18,19]. As a result, only a minor part of those papers deals with the anchors’ shear strength and with post-installed anchors.

Moreover, the phenomena that have been investigated for cast-in and post-installed shear anchors are premature failure modes, i.e., concrete edge failure, concrete splitting, anchor failure, concrete breakout, concrete pry-out failure, cyclic load failure, collapse due to prying actions [20,21,22,23,24,25,26,27,28,29,30,31,32,33,34,35,36,37,38,39,40,41,42,43,44,45,46,47,48,49,50,51,52,53,54,55,56]. Conversely, strength dictated by the concrete that surrounds the anchors by a pure shear mechanism has not been investigated, although this failure mode is the one that provides the greatest strength, so it should be the aim of anchor design as long as the geometric conditions allow it.

Eventually, shear anchors post-installed into hardened concrete have received less attention by research communities.

Another lacuna in the literature is that almost all the studies have investigated the post-installed anchors from the experimental and numerical perspective [57,58,59,60,61,62,63,64,65,66], while no analytical modeling has been carried on. Consequently, the models that can be borrowed from literature are either empirical or consists of the application of the finite element method, while the literature includes no closed-form equation model to predict the shear strength of an anchor or an anchor group post-installed into concrete.

Ultimately, not one of the papers that are present in the published literature furnishes an analytical model to calculate the shear strength of anchors post-installed into hardened concrete, with adequate clearance to the edges.

Anchors intended for use in concrete are dealt with by many codes, requirements, specifications, standards, provisions, and test methods, edited by organizations for technical assessment [67,68,69,70,71,72,73,74,75,76,77,78,79,80,81,82,83,84,85].

Some of the above documents are entirely devoted to anchors, such as [67], which furnishes the 1997 state-of-the-art on anchorage to concrete, [68,69,73], which depict a general picture about anchorages, [74,75,76,84], which define standardizations for testing and using anchors, and [85], which deals with anchors for non-structural components. However, again, those documents are mainly devoted to extraction strength, cast-in anchors, and premature failure modes. As a results, none of those documents provide an analytical model to predict the shear strength of anchors post-installed into concrete that do not fail by premature failure modes.

More specifically, shear strength of anchors post-installed into concrete that fail by pure shear (and not by a premature failure mode) is touched upon in Chapter 17 of American Concrete Institute (ACI) 318-14 [79]. In fact, that chapter includes design provisions for adhesive anchor systems, both cast-in and post-installed. Nonetheless, the problem is not tackled analytically but experimentally. The corresponding testing criteria are defined in ACI 355.4 [84].

Another important document that mentions shear strength of anchors post-installed into concrete that fail by pure shear is [80]. Although not totally devoted to anchors, however, this document includes an appendix (Appendix B—Anchoring to concrete) that presents one of the most used approaches to anchor safety assessment. However, existing prediction equations in ACI Committee 349 (i.e., [80]) largely underestimate the shear strength for small-sized anchors and largely overestimate the shear strength for medium and large-sized anchors [41]. That inaccuracy is the result of the fact that the formulation of [80] is experimental, not analytical.

In conclusion, no analytical model can be borrowed, not only from scientific literature, but also from structural codes to reliably predict the shear strength of post-installed anchor groups [86].

There are instead reports (instructions) edited by producers and manufactures devoted to giving technical provisions, which include formulas for predicting the shear strength of anchors post-installed into concrete structures. Some of those formulas are implemented into software for practitioners. Unfortunately, that software and the predictions of those formulas are unrealistic, as shown in Section 9 of this paper.

As a result, the designer who wants to use shear anchors is obligated to perform on-site tests to directly measure the shear strength of the designed anchors. Unfortunately, measuring the shear strength of an anchor is labor intensive, and even more so for an anchor group. The reason is that the reaction of the shear force applied to the anchor is not directly resisted by the concrete structure (as it occurs in the pull-out test) but must be resisted by the concrete structure by means of a specific test set-up. In addition, on-site tests must be numerous in order to be statistically significant.

The testing procedure performed by the designer has to comply with codes or instructions. Namely, the performance of post-installed anchors in concrete must be evaluated by a testing procedure that is recognized and accepted. A popular testing procedure is that presented in [84]. That document prescribes testing programs and evaluation requirements for post-installed mechanical anchors intended for use in concrete under the design provisions of ACI 318 [79]. In that document, criteria are prescribed for determining whether anchors are acceptable for use in uncracked concrete only, or in cracked as well as uncracked concrete. Performance categories for anchors are also established, as are the criteria for assigning anchors to each category. The anchor performance categories are used by ACI 318 [79] to assign capacity reduction factors and other design parameters.

However, the reality can be even more demanding. If the test results are lower than the expected strength, the design must be changed, which entails serious problems for the designer, the client (or the owner of the building), and construction activity, since design changes must be carried out at the construction site stage.

It was due to exactly such a problem, found in his professional activity, that the author of this paper set up this research activity. This activity includes both single anchors and anchor groups, post-installed into either concrete or masonry. This paper is exclusively devoted to anchor groups post-installed into hardened concrete, while the other research topics are or will be published in other papers.

Ultimately, this paper provides an analytical (closed-form) mathematical model for predicting the shear strength of an anchor group post-installed into a hardened concrete whose compressive strength is known, far from the edges. The shear strength predicted by this model must be compared to the shear strength of the anchor group dictated by the metal of the anchors and by the distance from the edges. The actual shear strength of the anchor group is the lowest of those strengths.

## 2. Reference Structure and Scope of the Research Work

The present paper deals with post-installed anchors intended for use in concrete. The anchors form an anchor group, which transmits loads to or receives loads from a structural assembly, external to the surface of the concrete, called “attachment”.

The anchor group that is dealt with is sufficiently far from the edges of the concrete structure, so that the strength does not depend on the distance from the boundaries.

The author specifies that, since he is not enthusiastic about invention and deployment of new acronyms, this paper does not use any acronym.

Each anchor of the group is subjected to a transverse force applied to the end that protrudes from the concrete surface, i.e., a force orthogonal to the anchor. That force is called “shear force” and is denoted by the symbol *V_a_*. The sum of the shear forces *V_a_* that are applied to the anchors of the group is denoted by *V_g_*. Research activity is devoted to analytically predicting the maximum (ultimate) value that the shear forces *V_g_* can reach, which is called “shear strength” of the anchor group and is denoted by the symbol *V_gu_*.

The attachment that is here considered does not transmit bending moment to the anchor group, which implies that the connection between the anchor group and the attachment must be a hinge. That in theory. In reality, an attachment always transmits some bending moment together with the shear force. In fact, first, a forces *V_a_* is ever eccentric to the concrete surface, which implies some bending moment in the embedded anchor sections. Second, a hinge is never a pure pin, which implies some bending moment acting on the anchor group. Third, there is always some prying action, which implies further bending moment acting on the anchor group.

The bending moment acting on the anchor group is totally different than the bending moment acting on a single anchor. Actually, the latter has the same effect as the shear force—namely, the bending moment acting on a single anchor adds contact pressures to those due to the shear force. So, the bending moment acting on a single anchor does not modify the resisting mechanism of the anchor, which continues to be provided by contact pressures exchanged between the concrete and the embedded system. The model presented here accounts for the eccentricity of the shear forces *V_a_* with respect to the concrete surface (i.e., that eccentricity is one of the data). Namely, the model reproduces the bending moment induced by that eccentricity.

On the contrary, the bending moment acting on the anchor group (i.e., the former) gives rise to a couple composed of a compressive force, which induces compressive contact pressures on the concrete surface, and a tensile force, which induces tensile axial forces in some anchors of the group. While the compressive contact pressures play no role in the shear strength, the tensile force impacts on the shear strength of the anchor.

More specifically, tensile force in an anchor induces longitudinal shear stresses at the interface between concrete and anchoring material (or anchor), whose ultimate resultant is the extraction force (pull-out resistance).

Eventually, the bending moment acting on the anchor group due to the fact that the hinge is not a pure pin and due to some prying action, implies a resisting mechanism of the anchor group that combines shear strength and axial strength.

Non only does the prediction of the extraction force (pull-out resistance) require a specific model, but also there is a substantial interaction between the shear strength and the pull-out strength. The effects induced by the shear force cannot hence be superimposed to the effects induced by the axial force. So, that anchor must be modeled under combined shear and axial loading.

However, the point is not the prediction of the strength when the anchor group is simultaneously subjected to shear force and substantial axial force, but the optimal design of an anchor group. The interaction between shear force and axial force drastically reduces the strength of an anchor. Thus, design should minimize the bending moment acting on the anchor group. Namely, the connection should be designed to behave as close as possible to a pin and to reduce prying action as much as possible. Ergo, an optimally designed connection transmits no more than moderate bending moment to the anchor group, so that the axial force introduced into every anchor is marginal.

This paper refers to optimally designed connections. Accordingly, the anchors that this paper is devoted to, are subjected to substantial shear force and marginal axial force. Ultimately, the axial force in each anchor of the group that is dealt with does not interact with the shear force, so it can be neglected.

For the sake of clarity, this paper assumes that each anchor is subjected to a transverse force *Va* (i.e., individual shear force) applied to the end that protrudes from the concrete surface, but no parallel force (i.e., no axial force is applied to the anchor). It follows that the anchors subjected to substantial axial force together with the shear force is not represented by this model.

For the same reason, the attachment that is considered here does not transmit axial force, which however is quite a normal condition.

It is noteworthy to mention that the collocation “shear force”, used in this paper (including the title) refers to the external force acting on an anchor, while the internal forces in an anchor are shear force and bending moment.

### 2.1. Adhesive Anchor and Mechanical Anchor

Anchors can be adhesive or mechanical. This paper refers to both types of anchors.

The insertion of an adhesive anchor into hardened concrete first requires drilling a hole into the concrete. Drilled holes shall be perpendicular to the surface of the concrete member (a rotary hammer drill can be used to drill all holes). All holes must be cleaned using a stiff brush (a stiff bottle brush to loosen as much dust as possible) and then vacuumed using an industrial vacuum cleaner (with adequate nozzle) to remove dust from the sides and bottom of hole. This procedure must be continued until a gloved finger rubbed on the walls came out dust-free.

Then, post-installation goes on filling the hole with an anchoring material (the adhesive must be placed under favorable conditions, in particular the temperature), and inserting the anchor into the hole, which implies that the anchoring material in excess is expelled outside (excess adhesive must be removed from the concrete surface).

Post-installation is complete when the anchor touches the bottom of the hole. Anchors must be cured at adequate temperature and number of days before being loaded.

This type of anchor is called “adhesive anchor” after the anchoring material. For both structural and non-structural anchors, it is important to ascertain that the adhesive anchor system that is selected reliably performs in rigorous or adverse jobsite conditions.

If the anchoring material is a resin, the drilled hole must be tight to the anchor. In fact, resins are relatively soft and, if the gap between anchor and drilled hole were not small, the layer of resin would result rather thick, which would imply that the anchor will undergo relative movements to the drilled hole. If conversely the anchoring material is made of mortar, the drilled hole can be appreciably greater than the anchor.

Alternatively, the anchor can be fixed to the concrete without anchoring material. This type of anchor is called “mechanical anchor” after the way it is bonded.

In this case, the anchor achieves resistance either by taping its own hole as the screw is driven into the concrete or by means of expansion against the concrete or against a sleeve (this procedure includes drilling a hole slightly larger than the sleeve).

The way a mechanical anchor is post-installed implies that the drilled hole is as wide as the anchor.

This paper refers hence to anchors that achieve resistance by bonding with adhesives and anchors that are bonded to the concrete without any adhesive. Actually, the pull-out mechanism is not involved, as the anchor resists only transverse forces. So, the way the anchor is bonded to the concrete plays no role in the strength of the anchor.

The whole system—anchors, anchoring material, drilled holes, and concrete that surrounds the drilled holes—is here called “anchorage”.

### 2.2. Specific Features of the Post-Installed Anchor

Each anchor that composes the anchor group this paper refers to is a metal element post-installed into a hardened concrete member and used to transmit applied loads. Typically, anchors are made of steel (carbon, stainless, galvanized, or titanium steel).

Every anchor is perpendicular to the surface of the concrete member, so the external force *V_a_* is parallel to the surface of the concrete. If the anchor were not perpendicular to the concrete surface or the external force were not parallel to the concrete surface, such anchor would be subjected to axial force together with the shear force.

The anchors of the group have the same length and embedded depth. The total length of the anchor is denoted by *L*. One end of the anchor is embedded into the concrete, while the other end protrudes from the concrete, in order to allow the anchor to be connected to the attachment. Each shear force *V_a_* is applied at the protruding end, whose distance from the concrete surface is denoted by *e*. The embedded depth of the anchor is hence *L* − *e*. The length *e* depends on the anchor and the attachment.

An anchor installed into a hardened concrete member (post-installed anchor) is different than an anchor installed before placing concrete (cast-in anchor). Typically, the latter has a hook at the embedded end, and the embedded part includes nuts, washers, and/or plates. Moreover, the member the anchor is cast into includes specific bars and stirrups that embrace the anchor.

A post-installed anchor requires drilling a hole into the concrete, which prevents the embedded end from being hooked and any attachment from being placed along the shaft of the anchor. Consequently, the post-installed anchor is a straight shaft (shank) with no nut, washer, or plate attached to the shaft. Moreover, the concrete member that the anchor is post-installed into does not include any reinforcement devoted to transferring the shear from the anchor to the member, as the attachment is a load not included in the original design.

Ultimately, the post-installed anchor this paper refers to, cannot rely on any strengthening component in addition to the straight shaft embedded into the concrete.

Each anchor should be installed into uncracked concrete. If the concrete is cracked, the anchors should have adequate clearance from the cracks, so that the failure mode is not dictated by cracking existing prior the installation. Where this criterion cannot be met, the shear strength of the anchor is lower than that predicted by this model, but however only moderately [7,12,16,65].

Needless to mention, the cracks that are referred to just above are those which have occurred before installing the anchors, while the cracks that occur in the concrete that surrounds the anchors when *V_g_* reaches high values are considered by the model, as these cracks belong to the main events that lead to shear failure (i.e., at the ultimate the concrete is cracked, as any RC member subjected to shear and/or bending).

## 3. Anchor Group: Definitions and Nomenclature

Literally, a shear anchor group, which is the subject that this paper focuses on, is a number of anchors that bear shear forces *V_a_*, which sum up together giving rise to the resultant force *V_g_* transmitted between the anchor member and the attachment. However, the reason why the anchors are dealt with as a group instead of as individual elements independent of one another is that relatively close anchor spacings can generate some structural effects due to interaction between anchors.

Appendix B of [80] (Anchoring to concrete) defines an anchor group as a number of anchors of approximately equal effective embedment depth with each anchor spaced at less than three times its embedment depth from one or more adjacent anchors. This paper does not adopt that definition, because this research demonstrated that it does not capture the anchor group effect.

Typically, the anchors of a group are collected by a steel plate, which is connected to the attachment (e.g., to a beam) that the anchor group connects to the concrete member. Accordingly, the anchor group transmits the resultant force *V_g_* from the attachment to the concrete member, and vice versa the anchor group transmits the resultant force *V_g_* from the concrete member to the attachment (through the plate). The resultant force *V_g_* is the sum of *N* force *V_a_* transmitted across the *N* anchors of the group.

Design of an anchor group shall start defining the largest circumference within which the anchors can be placed satisfying all the design geometric restraints, the design constraints, and the design prescriptions. That circumference drawn onto the concrete surface is called “circumscribing circumference”. The diameter of that circumference is denoted by *D* and its center by *C*.

**Figure 1:**
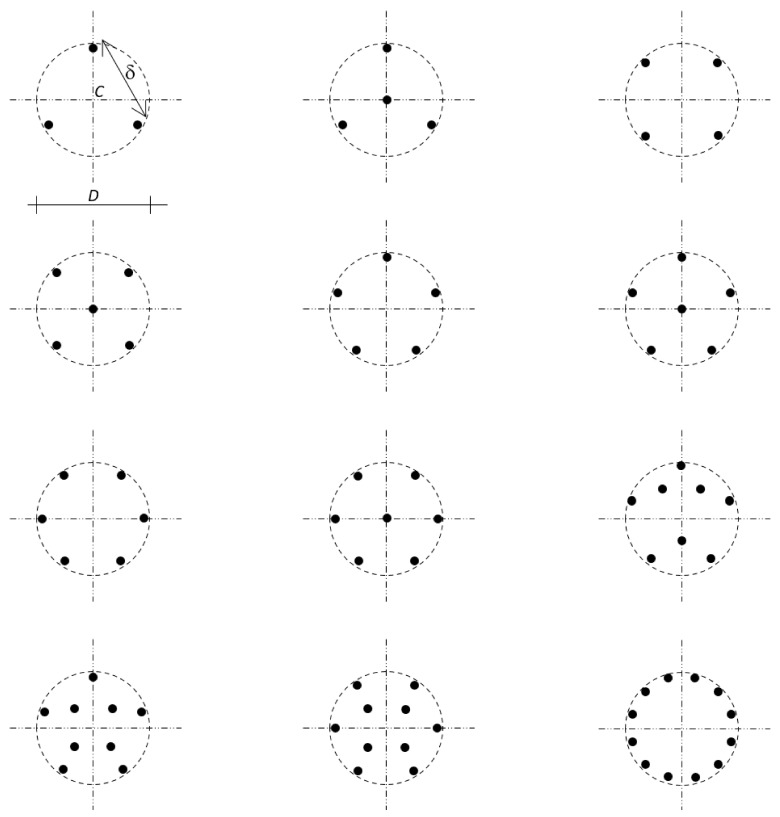
Possible patterns. Three-, four-, five-, six-, seven-, eight-, nine-, ten- twelve-anchor patterns. Each pattern is circumscribed by a circumference of diameter *D* and center *C* (these symbols are shown by the pattern 1-1 together with the spacing δ). The circumscribing circumference defines a cylinder of diameter *D* and height *L* − *e*, called circumscribing cylinder. The shear force acting on an anchor group is applied at the center *C* of the circumscribing circumference (which lies on the concrete surface).

The anchors shall be placed equally spaced on that circumference, so as to be axially symmetric around the center *C*. Then, if necessary, the pattern shall be integrated by anchors inside the circumference, placing these anchors approximately axially symmetric around the center. Figure 1 shows some anchor patterns that meet this criterion.

The diameter of an anchor is denoted by ϕ. The number and spacing of anchors are denoted by *N* and δ, respectively (Figure 1). The embedded length of an anchor has been defined as *L* − *e*. The embedded length *L* − *e*, diameter ϕ, number *N*, spacing δ, and anchors’ pattern shall obviously be defined based on the shear strength that must be achieved.

This research focuses on anchor groups that are placed in the above-described fashion. Differently than [80], the spacing δ is not included into the definition that is here adopted for “anchor group”. On the other hand, however, δ determines three types of behaviors, as explained in Section 4.1. So, the role played by the spacing δ in the shear strength is crucial.

Further than be arranged in the above-mentioned fashion, the anchor groups that this research focuses on are assumed to be properly designed and installed, which implies that the anchors must be spaced not less than 2.0 diameters on center. Namely, the center-to-center spacing is δ ≥ 2·ϕ, otherwise the drilled holes triggers cracks in the concrete between the anchors.

**Figure 2:**
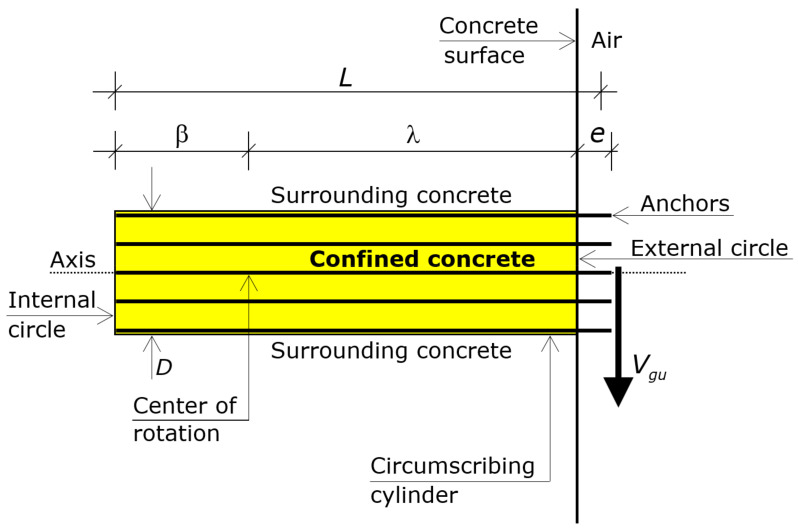
Longitudinal cross-section of the anchor group: circumscribing cylinder (diameter *D* and height *L* − *e*). The figure shows its lateral surface, which is shadowed, together with its external and internal circles. The figure also shows the anchors (pattern 4-2 of Figure 1) and the center of rotation, which lies on the axis of the cylinder. The concrete within the cylinder is confined. The “surrounding concrete” is the material that dictates the shear strength of the anchor group.

Modeling refers to the solid generated by the translation of the circumscribing circumference along the axis that passes through the center *C* and is perpendicular to the external concrete surface. That translation occurs from *C* to the embedded ends of the anchors of the group (Figure 2).

That solid is a cylinder, which is called “circumscribing cylinder”. The height of the circumscribing cylinder is hence *L* − *e*.

The boundary circles of the cylinder lie on the external surface of the concrete and at the embedded ends of the anchors, respectively. The former is called external circle (its boundary is the circumscribing circumference) and the latter is called internal circle (Figure 2).

The force *V_gu_* acts through the axis of that cylinder, at a distance *e* from the center *C* of the external circle (i.e., eccentric by *e* about the circumscribing cylinder).

**Figure 3:**
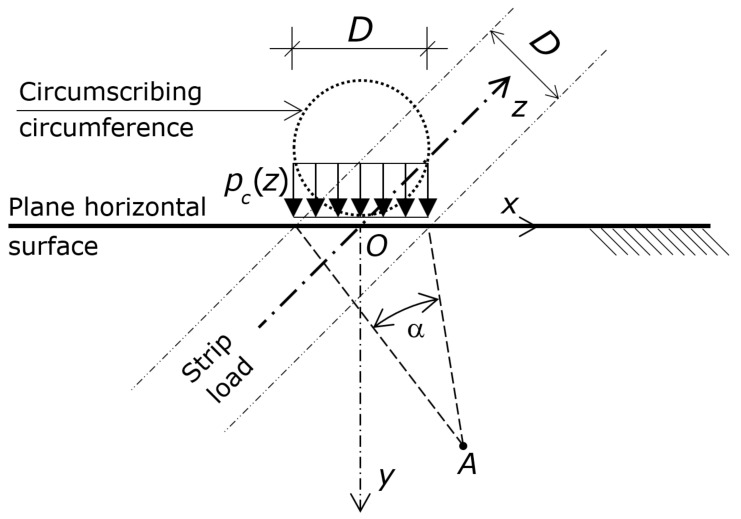
Cartesian coordinate reference system *x*-*y*-*z*, and angular α-coordinate of the generic point *A*. The origin of the Cartesian coordinate system is on the concrete surface, the *x*-axis is tangent to the external circle and orthogonal to the shear force *V_g_*, the *y*-axis is along the shear force *V_g_*, and the *z*-axis is parallel to the axis of the circumscribing cylinder. The figure shows the compressive stresses *p_c_*, which act on a strip whose width is along the *x*-axis and is tangent to the circumscribing cylinder. The compressive stresses *p_c_* acting at a given *z* are constant along the width *D* (i.e., do not vary along the axis *x*).

Two coordinate reference systems are used (Figure 3). The first one is a cartesian *x*-*y*-*z* system, with origin on the surface of the concrete, which is mainly used to identify the depths (by the axis *z*, which is parallel to the axis of the circumscribing cylinder).

The second one is the angle α, which is used to define the positions on the *x*-*y* plane at a given depth *z*. The coordinate α of a point *A* at a given *z* is the angle between two radii from *A* that pass through the edges of a segment that is tangent to the circumscribing cylinder at the origin of the Cartesian coordinate system, orthogonal to the shear force *V_g_*, and having length *D*. Of course, a given α does not identify a single point (apart from α = π), but a curve (Figure 3).

The anchor groups that are modeled in this paper have adequate clearance from the boundaries (edges) of the structure, so that failure is not dictated by the distance of the anchors from the edges. Ergo, the shear strength is not reduced by edge effects.

Where clearance from the edges is not large, the shear strength obtained from this model must be compared to that obtained from formulations that allow for edge effects [80,81,82,83,84,85]. The actual shear strength is the lowest one.

Ultimately, this paper models anchors that form an anchor group, post-installed into uncracked concrete, however installed and bonded, subjected to pure shear forces applied at the external ends, which do not suffer from interaction with the edges of the concrete member, whose failure is dictated by the concrete that surrounds the anchors.

Namely, this paper studies anchor groups composed of either adhesive or mechanical anchors, post-installed in hardened concrete, with no global bending moment acting on the anchor group (no axial force in the anchors), which fail by shear load mechanism, whose strength does not depend on the distance from the edges (no effects of proximity to edges), in which cracks trigger only approaching the ultimate shear force (whereas if the concrete is already cracked, the strength is only moderately lower).

This paper uses the term “concrete” because the contribution that the reinforcement provides shear strength with is usually marginal. Moreover, that contribution cannot be generalized for post-installed anchors. Nevertheless, the members that the anchors are post-installed into are typically made of reinforced concrete.

## 4. Preliminary Numerical Analyses

The author performed numerical non-linear analyses of an exhaustive variety of anchor groups embedded into concrete and subjected to shear forces, in order to understand the mechanical behavior and failure modes.

Anchors and anchoring material were modeled as linear elastic, using the relevant elasticity moduli.

In the case of adhesive anchors, the interface between an anchor and the surrounding anchoring material was modeled as a bilateral contact. The interface between anchoring material and surrounding uncracked concrete was modeled as a bilateral contact, while the interface between anchoring material and surrounding cracked concrete was modeled as a unilateral contact.

In the case of mechanical anchors, the interface between anchor and surrounding uncracked concrete was modeled as a bilateral contact, while the interface between anchor and surrounding cracked concrete was modeled as a unilateral contact.

The stress–strain relationships of concrete was modeled using the following function between the compressive stress σ_c_ and the compressive strain ε_c_, in which compressive stresses and strains are positive

(1) $$\sigma_{c}\;=\;\frac{\frac{E_{c}}{E_{c1}}\cdot \frac{\varepsilon_{c}}{\varepsilon_{c1}}\;-\;{\left(\frac{\varepsilon_{c}}{\varepsilon_{c1}}\right)}^{2}}{1\;+\;\left(\frac{E_{c}}{E_{c1}}\;-\;2\right)\cdot \frac{\varepsilon_{c}}{\varepsilon_{c1}}}\cdot f_{cm}$$

where *f_cm_* is the concrete compressive strength, expressed in N/mm^2^.

The strength *f_cm_* and the strain ε_c1_ of Equation (1) account for the confinement action that the anchor pattern provides the concrete of the circumscribing cylinder with, and for the confinement action that the biaxial stress state provides the concrete surrounding the circumscribing cylinder with. It is to note that the former confinement is drastically greater than the latter, although the latter is not negligible.

The increased strength *f_cm_* of Equation (1) was estimated by the following equations, in which compressive stresses are positive:

(2) $$f_{cm}\;=\;f_{c}\cdot \left(1.0\;+\;\frac{5.0\cdot \sigma_{2}}{f_{c}}\right)\;\;\;\;\;\mathrm{for}\;\;\;\sigma_{2}\;\leq \;0.05\cdot f_{c}$$

(3) $$f_{cm}\;=\;f_{c}\cdot \left(1.125\;+\;\frac{5.0\cdot \sigma_{2}}{f_{c}}\right)\;\;\;\;\;\mathrm{for}\;\;\;\sigma_{2}\;>\;0.05\cdot f_{c}$$

where *f_c_* is the compressive strength of concrete under a uniaxial state of stress.

The member that the anchors are embedded into is existing, which entails that *f_c_* has to be defined performing in-place tests, considering the necessary factors in planning in-place tests, as well as considering the specified design strength, if available.

A variety of techniques are available for estimating in-place strength of concrete. The methods to estimate in-place strength of concrete include rebound number, penetration resistance, pullout, break-off, ultrasonic pulse velocity, maturity, and cast-in-place cylinders. Adequate procedures are then needed to derive compressive strength from in-place results, which include using appropriate statistical techniques to interpret test results.

The stress σ_2_ of Equations (2) and (3) is the lowest compressive principal stress. Namely, σ_2_ is the smallest confining stress of the triaxial stress state.

The increased strain ε_c1_ of Equation (1) was estimated by the following equations, in which compressive strains are positive:

(4) $$\varepsilon_{c1}\;=\;0.002\cdot {\left(\frac{f_{cm}}{f_{c}}\right)}^{2}$$

The elasticity modulus *E_c_* of Equation (1) is the tangent modulus given by Equation (5), with both *f_cm_* and *E_c_* in N/mm^2^.

(5) $$E_{c}\;=\;22000\cdot {\left(\frac{f_{cm}}{10}\right)}^{0.3}$$

In (5), *f_cm_* is the increased strength given by either (2) or (3).

The elasticity modulus *E_c_*_1_ of Equation (1) is *E_c_*_1_ = *f_cm_*_/_ε_c1_, with both *f_cm_* and *E_c_*_1_ expressed in N/mm^2^, in which *f_cm_* is given by either (2) or (3), and ε_c1_ by (4).

For the descending part of the stress-strain function, Equation (1) is valid only for values of σ_c/_*f_cm_* ≤ 0.5. The strain ε_cL_ at σ_cL_ = 0.5·*f_cm_* is given by Equation (6).

(6) $$\varepsilon_{\mathrm{cL}}\;=\;\varepsilon_{c1}\cdot \left\{\frac{1}{2}\cdot \left(\frac{1}{2}\cdot \frac{E_{c}}{E_{c1}}\;+\;1\right)\;+\;{\left[\frac{1}{4}\cdot {\left(\frac{1}{2}\cdot \frac{E_{c}}{E_{c1}}\;+\;1\right)}^{2}\;-\;\frac{1}{2}\right]}^{0.5}\right\}$$

For strains ε_c_ > ε_cL_ the descending branch of the σ_c_ − ε_c_ function was described using Equation (7):

(7) $$\sigma_{c}\;=\;f_{cm}\cdot {\left[\left(\frac{\zeta }{\lambda }\;-\;\frac{2}{\lambda^{2}}\right)\cdot {\left(\frac{\varepsilon_{c}}{\varepsilon_{c1}}\right)}^{2}\;+\;\left(\frac{4}{\lambda }\;-\;\zeta \right)\cdot \frac{\varepsilon_{c}}{\varepsilon_{c1}}\right]}^{-1}$$

with

(8) $$\zeta \;=\;\frac{4\cdot {\left[\lambda^{2}\cdot \left(\frac{E_{c}}{E_{c1}}\;-\;2\right)\;+\;2\cdot \lambda \;-\;\frac{E_{c}}{E_{c1}}\right]}_{}}{{\left[\lambda \cdot \left(\frac{E_{c}}{E_{c1}}\;-\;2\right)\;+\;1\right]}^{2}}$$

and with

(9) $$\lambda \;=\;\frac{\varepsilon_{\mathrm{cL}}}{\varepsilon_{c1}}$$

Compressive failure of concrete is often a discrete phenomenon, i.e., there is a fracture region of limited width, in which compression strains are concentrated [87].

For practical reasons and due to lack of sufficient experimental data, these strain concentrations generally are smeared, as has been done in Equations (1)–(7). As a consequence, the descending branch of the stress-strain relation in compression is influenced by the length of the member subjected to compression.

Due to such uncertainty and highly variable performance, the descending portion of the stress-strain relation is considered as an envelope to all possible stress-strain relations of concrete which tends to soften as a consequence of concrete micro-cracking. In view of that, the concrete constitutive law of Equation (7) can be used either imposing a limit on ε_c_ or without imposing any limit on ε_c_. That limit is the crushing strain of concrete, which is herein denoted by ε_cu_. In the former case, the contribution to the anchor’s shear strength provided by the concrete depends on ε_cu_, while in the latter case does not depend on ε_cu_.

The increased strain ε_cu_ (i.e., the ultimate strain that accounts for the confinement action) was estimated by the following equation:

(10) $$\varepsilon_{\mathrm{cu}}\;=\;0.0035\;+\;0.2\cdot \frac{\sigma_{2}}{f_{c}}$$

The results of the non-linear analysis demonstrated that the shear strength of an anchor group depends slightly on ε_cu_. On that account, Equation (7) was used without any limit on ε_c_, i.e., without applying ε_cu_.

Tensile failure of concrete is always a discrete phenomenon, as compressive failure. Differently than compressive failure, however, tensile failure has been extensively studied by fracture mechanics and the results that were obtained allow cracks to be accurately modeled. Furthermore, smearing the strain concentrations would yield lower accuracy than for compressive failure [88].

To describe the tensile behavior, thus, a stress-strain diagram was used for the uncracked concrete, and a stress-crack opening diagram for the cracked section. More specifically, for uncracked concrete subjected to tension, a bilinear stress-strain relation was used, composed of two ascending branches with decreasing slope. For a cracked section, a bilinear stress-crack opening relation was used, composed of two descending branches with decreasing negative slope.

A general non-linear finite element model was then prepared, which incorporated the above-described constitutive laws and reproduced each component using the relevant geometry and mechanical properties. In so doing, accurate simulations of the responses of anchor groups to shear forces were obtained.

If on one hand the results of that model are accurate and comprehensive (as proven by comparison to experimental results borrowed from literature), on the other hand, the numerical model is not easy to use in daily practice. So, the model cannot be proposed for practitioners’ activity. In fact, to run the numerical model, and to understand and visualize the data outputs require spending amounts of time and effort that are excessive. Research was directed towards providing a tool for daily practice of the structural designer that retains the accuracy of the non-linear finite element model. To that end, the non-linear finite element model was used to simulate a sufficiently comprehensive series of cases, in order to identify general behaviors shared by all the anchor groups. Those general behaviors allowed an analytical model to be developed.

### 4.1. Basic Behaviors of an Anchor Group Embedded into Concrete

The analyses performed using the above-described non-linear finite element model showed that all the anchor groups have the same type of ultimate behavior and the same failure mode, which allowed mechanical assumptions to be established.

The key to understanding the ultimate behavior is to focus on the circumscribing circumference (Section 2), whose diameter is *D* (Figure 1), and on the circumscribing cylinder (Figure 2), whose height is *L* − *e*, and whose two bases are the circle at the concrete surface (external circle whose boundary is the circumscribing circumference) and the circle where the embedded ends of the anchors lie on (internal circle).

The circumscribing cylinder includes concrete, anchors, and anchoring material (unless the anchors are fixed to concrete without anchoring material).

Numerical modeling results show that the anchors can substantially confine the concrete. The level of confinement depends on how much the anchors exploit the area of the circumscribing circumference. The results pointed out three ranges of behavior depending on the spacing δ of the anchors and embedded length *L* − *e*. Those ranges are defined by the limits reported immediately below, expressed in mm.

close spacing: δ ≤ 2.8·ϕ·(*L* − *e*)/160 (11)

intermediate spacing: 2.8·ϕ·(*L* − *e*)/160 ≤ δ ≤ 3.4·ϕ·(*L* − *e*)/120 (12)

wide spacing: for δ > 3.4·ϕ·(*L* − *e*)/240 (13)

Close or intermediate spacings of the anchors provides the circumscribing cylinder (i.e., the concrete inside it) with a substantial level of confinement, which drastically increases its stiffness and strength, with respect to the stiffness and strength of the concrete that surrounds the circumscribing cylinder. That level of confinement makes the deformation of the circumscribing cylinder be negligible with respect to that of the surrounding concrete. Moreover, that level of confinement makes the concrete of the circumscribing cylinder be substantially stronger than that of the surrounding concrete.

**Figure 4:**
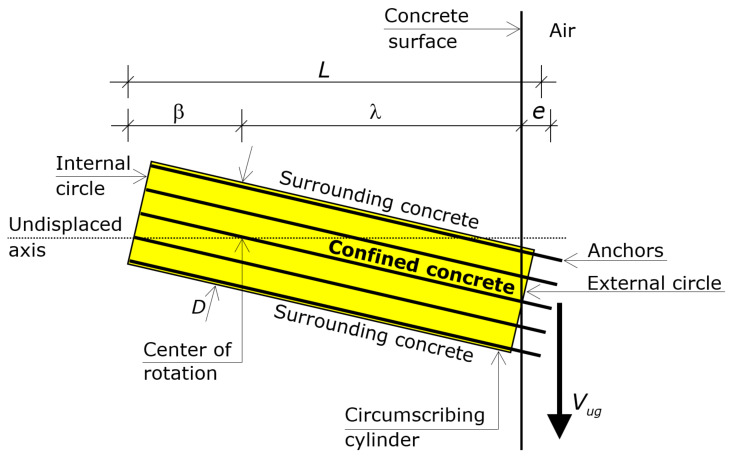
Failure mode of an anchor group with close or intermediate spaced anchors: the circumscribing cylinder, which is confined, rotates around a point located on its axis. Rotation causes the lateral surface to push against the surrounding concrete. Rotation also causes the lateral surface to pull the surrounding concrete, but that action induces negligible stresses. The figure also shows the position of the rotation center on the axis of the undisplaced circumscribing cylinder.

Eventually, by virtue of the confinement provided by the anchors for close or intermediate spacings (Figure 1 and Figure 2), the circumscribing cylinder can be assumed to behave as a rigid body up to failure—namely, that cylinder undergoes a rigid body motion up to the ultimate—while the surrounding concrete reaches substantial deformations (Figure 4).

By virtue of that confinement, hence, the surrounding concrete reaches strains much greater than the circumscribing cylinder, so that the ultimate shear force is dictated by the concrete that surrounds the circumscribing cylinder, while the concrete of the circumscribing cylinder reaches stresses far from the concrete crushing strength.

Eventually, the “group effect” consists of those drastic increases in stiffness and strength of the concrete circumscribed by the cylinder.

On the contrary, wide spacings of the anchors provide the circumscribing cylinder with marginal confinement, which does not appreciably increase its stiffness and its strength, with respect to the surrounding concrete. Thus, wide spaced anchors do not give rise to any group effect.

For close and intermediate spacings, hence, modeling can replace the anchors and the enclosed concrete with the circumscribing cylinder, whose strength and stiffness can be assumed to be infinite. That is, the circumscribing cylinder behaves as if it were a sole anchor of diameter *D* and embedded length *L* − *e*.

Ultimately, the concrete of the circumscribing cylinder (i.e., the concrete between the anchors) does not fail but dictates the strain profile that causes the failure of the surrounding concrete.

For an anchor group with wide spacing, each anchor behaves as an individual member, due to a marginal confinement action induced by the anchor group. Such confinement increases neither the stiffness nor the strength of the circumscribing cylinder. It follows that no anchor group effect occurs for wide spacing, and the shear strength depends on the concrete that surrounds each individual anchor. The shear strength of wide spaced anchors has therefore to be obtained for the model of the single (individual) anchor, whereas the model delivered here does not apply.

The analysis of the numerical results focused on the ratio between the shear strength of an anchor group and the sum of the shear strengths of each anchor of the group behaving as an individual anchor (i.e., single components without any anchor group effect). The three ranges that have been introduced above derive from those ratios.

For close spacing of anchors, that ratio is lower than unity. Accordingly, the impact of the anchor group effect on the ultimate behavior consists not only in a confinement but also in a reduction in the potential strength of the anchors. That is, the shear capacity of the anchor group is lower than the sum of the individual shear capacity of the anchors.

For intermediate spacing of the anchors, that ratio is regularly greater than unity, which means that the impact of the anchor group effect on the ultimate behavior consists of an increase not only in the stiffness but also in the potential strength of the anchors. That is, the shear capacity of the anchor group is often greater than the sum of the individual shear capacity of the anchors.

While the result obtained for close spaced anchors is intuitive, the result obtained for intermediate spaced anchors is counterintuitive. The reason of a ratio greater than unity lies in the fact that the anchors provide the concrete of the circumscribing cylinder with substantial confinement, at which concrete compressive strength and stiffness are enhanced. As a result, the strength is dictated by the concrete that surrounds the circumscribing cylinder, and not by the concrete that surrounds each individual anchor. At the same time, intermediate spacing implies that the number of anchors is not high, so that the sum of the individual strength of the anchors is not high too, in particular may be lower than the shear strength of the anchor group. Namely, the anchors do not completely exploit the area of the circumscribing circumference, which in turn implies that the anchor group effect boosts the shear capacity of the group.

For wide spacing of the anchors, that ratio is equal to unity, which means that the anchor group effect does not impact on the strength of the group. This is the logical result of the fact that such spacing does not provide the concrete with any appreciable confinement. So, wide spaced anchors cannot be called anchor group since no anchor group effect exists.

Since research activity was directed at investigating anchor group effect, hereinafter, the paper considers only close and intermediate spacings, whereas wide spacing enters the topic of the single anchor.

The rigid body motion of the circumscribing cylinder that results for close or intermediate anchor’s spacings is composed of a uniform translation parallel to the shear force and a rotation in the *y*-*z* plane. The uniform translation is slight and can be neglected compared to the linearly varying translations due to the rotation.

The rotation makes the cylinder translate parallel to the shear force. Translations of the points of the circumscribing cylinder are in proportion to the distance from the rotation center. The direction of the translation of a point depends on the side of the point with respect to the rotation center. In particular, the rotation causes the external circle of the cylinder to exhibit a translation in the direction of the shear force (positive direction) and the internal circle to exhibit a translation in the opposite direction (negative direction), as shown in Figure 4.

The distance of the rotation center from the external circle (surface) is denoted by λ and that from the internal circle (embedded ends) by β, where *L* = λ + β + *e* (Figure 2). The length λ translates in the direction of the shear force, while the length β translates in the direction opposite to the shear force (Figure 4).

The rotation generates both tensile and compressive strains in the concrete. The tensile strains induce tensile stresses, which cause concrete to crack. Cracking causes most of the tensile stresses to disappear. Eventually, the tensile strain and stresses, as well as the cracks, can be neglected, and the sole effect of the rotation that is here considered is the compressive stress state.

Made that assumption, the elaboration of the results from the numerical model aimed to define the compressive stresses in the concrete at the ultimate—i.e., when the anchor group reaches the shear strength (not at the rupture, which is reached with a softening behavior, so that the shear force at the rupture is lower than the shear strength).

In other words, the shear force *Vg* applied to the anchor group is balanced by the stresses acting on the lateral surface of the circumscribing cylinder. Thus, elaborations were directed at defining the stresses acting on the lateral surface of the circumscribing cylinder at ultimate.

**Figure 5:**
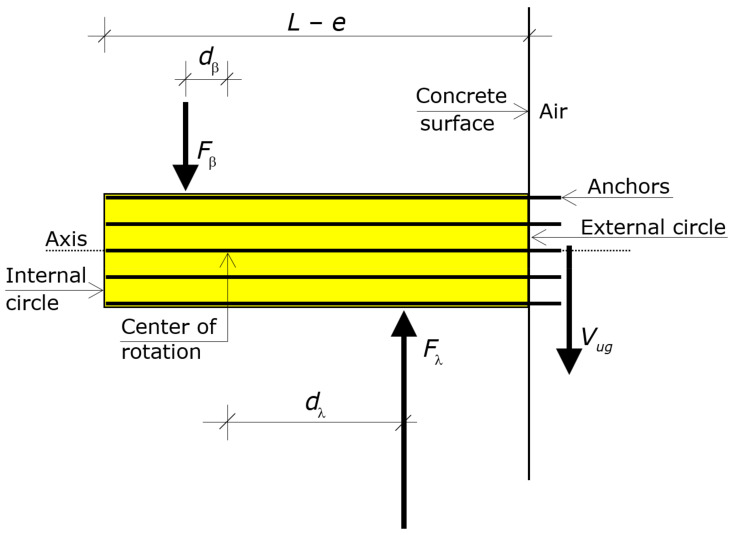
Internal forces *F*_λ_ and *F*_β_ acting on the circumscribing cylinder at ultimate and relevant application points, including the distances from the rotation center *d*_λ_ *d*_β_, respectively.

Elaborations of the numerical results allowed the stresses acting on the lateral surface of the cylinder to be replaced by two resultant forces acting on the lateral surface of the cylinder (Figure 5), while two equal and opposite forces act on the concrete adjacent to the circumscribing cylinder.

The first force is the stress resultant acting on the segment λ of the circumscribing cylinder at ultimate, whose direction is opposite to *V_g_*, which is denoted by *F*_λ_. The second force is the stress resultant acting on the segment β of the circumscribing cylinder at ultimate, whose direction is that of *V_g_*, which is denoted by *F*_β_ (Figure 5).

The force *F*_λ_ was proven to be, on average, equal to:

(14) $$F_{\lambda }\;=\;0.84\cdot {\left(\frac{f_{cm}}{33.0}\right)}^{0.11}\cdot D\cdot \lambda \cdot f_{cm}$$

where *f_cm_* is the maximum compressive stress tolerated by the concrete that surrounds the cylinder, expressed in N/mm^2^. It follows that the entire model requires using N and mm.

The distance of the application point of *F*_λ_ from the rotation center, which is denoted by *d*_λ_, was proven to be, on average, equal to:

(15) $$d_{\lambda }\;=\;0.58\cdot \lambda$$

Upon inspection of the numerical results, *F*_λ_ and *d*_λ_ were proven to differ marginally from their average value. Ergo, Equations (14) and (15) accurately estimate *F*_λ_ and *d*_λ_.

**Figure 6:**
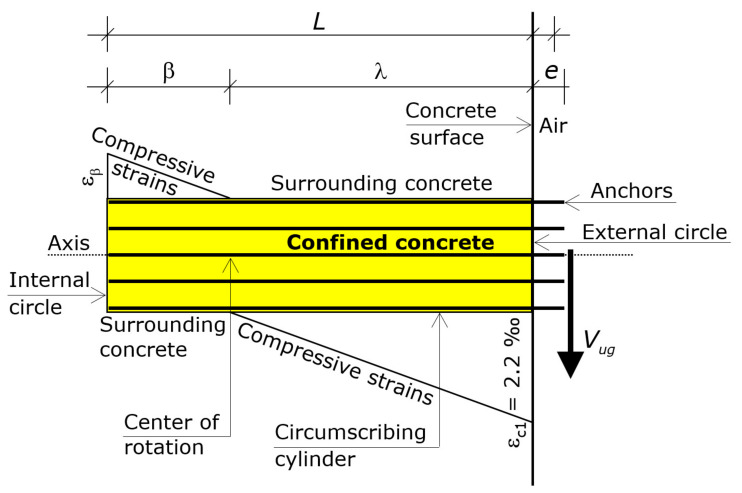
Profile of the compression strains at the interface between the circumscribing cylinder and the surrounding concrete. Strains vary along *z* but do not vary along *x*.

Since the behavior of the circumscribing cylinder is that of a rigid body, the compressive strain profile induced by *V_g_* along the cylindrical lateral surface (along *z*) is a straight-line, and along the width (along *x*) is uniform (Figure 6).

It is to note that the compressive strains at any z exist in the concrete that surrounds one semi-circumference of the circumscribing cylinder while do not exist in the concrete that surrounds the complementary semi-circumference, which exhibits either tensile strains or cracks.

In all the cases that were analyzed, the rotations at ultimate were found to differ marginally from the rotation that induces the compressive strain ε_c1_ of Equation (4) in the concrete that surrounds the external circle of the circumscribing cylinder. The rotation that induces ε_c1_ in the concrete surrounding the external circle provides hence an accurate estimation of the ultimate rotation.

Compression induced by the ultimate rotation in the concrete that surrounds the internal circle is elastic, since β is always much shorter than λ. That elastic compressive strain is denoted by ε_β_. The stress produced by ε_β_, which is elastic too, is denoted by σ_β_.

The compressive stress σ_2_ of Equations (2) and (3) is zero in the concrete that surrounds the external circle. Plugging that value into Equation (4), the compressive strain ε_c1_ turns out to be 2.2‰.

At ultimate, hence, the compressive strains on the lateral surface of the cylinder (Figure 6) range from ε_c1_ = 2.2‰ at the external surface to zero at the rotation center (segment λ), and from zero to a substantially elastic strain ε_β_ at the embedded ends of the anchors (segment β).

The resultant force of the compressive stresses acting on the segment β at ultimate, which is denoted by *F*_β_, is equal to the volume of the triangular prism whose sides are β, σ_β_, and *D*. The application point of *F*_β_ is the centroid of that prism.

Elaborations of the numerical results demonstrated that the stress state that exists in the concrete prior the installation of the anchor does not influence the shear strength of the anchorage.

Insight into the numerical results showed that σ_z_, τ_zx_, and τ_zy_ are marginal in the concrete that surrounds the circumscribing cylinder (the axes *x* and *y* are shown in Figure 3). It follows that the stress state in the concrete that surrounds the circumscribing cylinder is biaxial.

That conclusion agrees with another numerical results, which is that the shear strength of an anchor group with given embedment length *L* − *e* does not depend on the dimensions of the structure, as long as edge effects are prevented from occurring. In other words, how much the thickness (width) of the members surpasses *L* − *e* does not influence the shear capacity of a given anchor group.

#### Stress State in the Concrete That Surrounds the Circumscribing Cylinder

Insight into the compressive stresses induced by the circumscribing cylinder in the concrete that it pushes against allowed another general result to be achieved. Let us consider a circle of the circumscribing cylinder (i.e., a cross-section). The stresses in the concrete included into the square that circumscribes a circle are constant. That result agrees with the fact that, at a given *z*, the strain do not vary along *x* for—ϕ/2 ≤ *x* ≤ ϕ/2. Equations (14) and (15) are based on that result.

The circumscribing cylinder that presses against the surrounding concrete can hence be decomposed in the segment λ that pushes against the surrounding concrete in one direction and the segment β that pushes against the surrounding concrete in the other direction (Figure 4 and Figure 6).

Considering that the stress state is biaxial, the push of the segment λ or β against the surrounding concrete at a given *z* can be modeled by a semi-space (half-space) made of concrete. The surface of that half-space is a plane that passes through the origin of the Cartesian coordinate system and lies on the *x*-axis (Figure 3). Of course, the *z*-axis lies on that plane as well.

Considering that the stresses in the concrete included into the square that circumscribes a circle are constant and that the stresses due to loads different than the shear force *Vg* play no role, the push of the segment λ or β against the surrounding concrete at a given *z* can be modeled with a strip uniform load applied at the plane surface of the half-space. The width of this strip is *D*, while the magnitude of this load is that of the pressures *p_c_*(*z*).

In the end, the mechanical model [89,90,91,92] that best describes the stress state of the concrete that surrounds the circumscribing cylinder is the homogeneous, isotropic, weightless, linearly elastic semi-space (half space) with a plane surface that passes through the origin of the Cartesian coordinate system and is perpendicular to the shear force *V_g_*. The half space is loaded at that surface by a strip load of width *D* and magnitude *p_c_*(*z*). Although the half-space extends infinitely along the axes *x* and *z* (i.e., aside the embedded width and beyond the embedded ends), the strip load reproduces only the stresses at a given *z*, as the stress state is biaxial, so stresses do not depend on *z*.

According to that model, hence, the stress state at a given *z* induced by the contact pressures *p_c_*(*z*) is equal to the two-dimensional stress-state induced by *p_c_*(*z*) smeared along a strip of infinite length acting on a semi-space composed of the concrete that the *p_c_*(*z*) presses against. Consequently, the compressive stresses induced by either λ or β at a given abscissa *z* are simulated by a strip load whose value is that of the stresses at this *z* applied at the plane horizontal surface of the half-space (Figure 3). This model implies that the whole stress state in the surrounding concrete can be reproduced by as many half-spaces as the points on the axis *z* that are necessary to accurately describe the stress state. However, this model requires only the point on the axis *z* where the stress state reaches the maximum compression.

Equation (14) requires knowing the maximum compressive stress *f_cm_* tolerated by the concrete that surrounds the circumscribing cylinder, which is different than the strength of this concrete under a uniaxial state of stress *f_c_* because the stress state entails some confinement (although this confining action is drastically lower than that within the circumscribing cylinder).

Elaborations of the numerical results demonstrated that, in the concrete that surrounds the cylinder, the major principal stress σ1 and minor principal stress σ3 of the stress biaxial state, together with the principal stress in the third direction σ2 can be satisfactory approximated by the following expressions (compressive stress are assumed to be positive):

(16) $$\sigma_{1}\left(z\right)\;=\;\frac{p_{c}{\left(z\right)}_{}}{\pi }\cdot \left[\alpha \;+\;\sin\left(\alpha \right)\right]$$

σ_2_ = 0 (17)

(18) $$\sigma_{3}\left(z\right)\;=\;\frac{p_{c}{\left(z\right)}_{}}{\pi }\cdot \left[\alpha \;-\;\sin\left(\alpha \right)\right]$$

Equations (16)–(18) define the level of confinement of the concrete that surrounds the circumscribing cylinder, which in turn allows the maximum compressive stress *f_cm_* tolerated by that concrete to be defined. In so doing, Equation (14) can be used.

Equations (16) and (18) show that the dependance of σ_1_ and σ_3_ on *z* is dictated by *p_c_*(*z*), i.e., given *p_c_*(*z*), σ_1_ and σ_3_ do not depend on *z* (bi-axial stress state). Equation (17) shows that the principal stress σ_2_ is almost zero everywhere.

Equation (16) shows that the maximum σ_1_ occurs for α = π, i.e., at the middle of the circumscribing cylinder, and Equation (18) shows that σ_3_ reaches the maximum for α = π too, i.e., at the same point.

The principal stress σ_1_ causes the concrete to crush, the principal stress σ_3_ confines the concrete in the second direction, and the principal stress σ_2_ does not confine the concrete in the third direction. As a result, confinement is moderate. Thus, the effect of σ_1_ on crushing prevails over the effect of σ_3_ on confinement.

Ergo, *f_cm_* is dictated by the stress state σ_1_ = *f_cm_* (compression), σ_2_ = 0, and σ_3_ = *f_cm_* (compression), at the point at α = π.

For the above biaxial stress state, all the formulations that agree well with test data provide values of the biaxial concrete strength that only slightly exceed the following value of *f_cm_*:

*f_cm_* = 1.15·*f_c_* (19)

where *f_c_* is the strength of concrete under a uniaxial state of stress, which has been introduced about Equation (1). Eventually, Equation (19) is simultaneously an accurate and safe estimation of *f_cm_*.

In brief, the strength *f_cm_* to plug into (14) is not the uniaxial compressive strength of concrete *f_c_*, but is that of Equation (19)—namely, it is higher, as it benefits from some confinement provided by the biaxial state of stress. It is to note that *f_cm_* from (19) was used in the numerical model too (i.e., in Equations (1)–(5)).

Equations (16)–(18) were obtained from uncracked concrete. Nevertheless, in order to transmit those compressive stresses is sufficient that a circumference with diameter 1.5·*D* concentric to the cylinder be uncracked. That result agrees with [7,12,16,65].

## 5. Mechanical Assumptions

The results of the numerical analysis presented in Section 4 support and justify the following five mechanical assumptions, which allow the ultimate behavior of an anchor group to be analytically modeled, the collapse mechanism to be governed, and the shear strength to be predicted.

The assumptions refer to the circumscribing cylinder introduced in Section 3, whose boundary circles (which cover the cylinder) are one at the concrete surface (external circle, whose boundary is the circumscribing circumference) and the other at the embedded ends of the anchors (internal circle), respectively.

Depths are referred to the axis *z* of the Cartesian coordinate system (Figure 3), which defines the distances from the concrete surface.

As specified in Section 3, the anchors are installed at distances from the boundaries of the structure (edges) that make the boundary effect be negligible. Moreover, the anchors are installed so that no prying actions are present. As specified in Section 4.1, the anchors are installed in uncracked concrete (at least a circumference with diameter 1.5 times that of the circumscribed circumference should be uncracked).

The assumptions of the mathematical model are expressed by the following five statements.

**Assumption** **1.** *Stresses induced by loads different than the shear forces applied to the anchors’ ends that protrude from the concrete surface (whose sum is V_g_) are negligible*.

**Assumption** **2.** *The compressive strains at the interface between the lateral surface of the circumscribing cylinder and the surrounding concrete have a straight profile, which ranges from ε_c1_ = 2.2 ‰ at the external circle (z = 0) to zero at the rotation center (z = λ), and from zero to an elastic value ε_β_ at the internal circle (z = L − e)*.

**Assumption** **3.** *No tensile stresses act on the lateral surface of the circumscribing cylinder, and the shear stresses on the lateral surface are symmetric with respect to V_g_, so that the resultant force of the shear stresses is zero*.

**Assumption** **4.** *The magnitude of the resultant force F_λ_ of the compressive stresses acting on the lateral surface of the circumscribing cylinder is defined by Equation (14), in which f_cm_ is defined by Equation (19)*.

**Assumption** **5.** *The distance d_λ_ between rotation center and application point of F_λ_ is defined by Equation (15)*.

Assumption 1 allows the model to account only for the stresses induced by the anchorage. The position at which the anchor group is installed plays hence no role, as long as adequate clearance from the edges is guaranteed.

Assumption 2 is tantamount to say that the circumscribing cylinder has a rigid body rotation. The profile of the compressive strains in the concrete that surrounds the circumscribing cylinder is a straight line that starts from a known value of the strain and passes through the rotation center (Figure 6). The known value of the strain at the concrete surface is the strain that the rotation induces at the origin of the Cartesian coordinate system when the shear force reaches the maximum. That value was derived from the numerical analyses. Accordingly, when that strain is either lower or greater than ε_c1_ = 2.2‰ the shear force transmitted by the anchor group is lower than the ultimate value.

Once the position of the rotation center is known, hence, the strain at any *z* along the lateral surface can be calculated using direct proportionality.

Assumption 2 also establish that ε_β_ is elastic, which implies that the compressive stresses acting on the segment β are elastic too. Thus, the resultant force *F*_β_ is a known save for σ_β_ and β. Furthermore, the distance between the application point of *F*_β_ and the anchor’s rotation center *d*_β_ is known save for β.

Assumption 3 allows the model to neglect the tensile strains, and, in turn, the tensile stresses. This assumption is justified by the fact that the tensile strains cause the concrete to crack (the uncracked concrete around the circumscribing cylinder exhibits no more than small strains, which induce no more than marginal stresses). It follows that the strength of the anchor group stems from the compressive stresses acting on the lateral surface of the circumscribing cylinder.

Assumption 4 allows the compressive stresses acting on the segment λ of the cylinder lateral surface to be replaced by the resultant force *F*_λ_, which depend only on λ.

Assumption 5 allows the distance between the application point of *F*_λ_ and the anchor’s rotation center to be set equal to *d*_λ_, which depends only on λ.

## 6. Model That Predicts the Shear Strength of the Anchor

The five assumptions of Section 5 allow the non-linear numerical model (Section 4) to be replaced by an analytical model, which in turn allows the problem to be easily solved by closed-form equations. The analytical model retains the accuracy of the non-linear numerical model but not its complexity, since the five assumptions reduce the non-linear numerical model to the basic behaviors of an anchor group at the ultimate.

This section presents the analytical model that predicts the shear strength of an anchor group, which is the contribution made by this paper.

Given that the stresses acting on the segment β of the cylinder lateral surface are elastic (Assumption 2), the force *F*_β_ (resultant force of the compressive stresses acting on the segment β) can be obtained as a function of both β and the compressive stress at the internal circle σ_β_:

(20) $$F_{\beta }\;=\;\frac{D\cdot \beta \cdot \sigma_{\beta }\;}{2}$$

The force *F*_β_ is transverse to the anchors and acts on the lateral surface of the circumscribing cylinder. The direction of *F*_β_ is that of *V_gu_* (Figure 2). Obviously, an equal force with opposite direction acts on the concrete that surrounds the circumscribing cylinder.

Given that the profile of the stresses on β are elastic (Figure 6), the application point of *F*_β_ is at the distance *d*_β_ from the rotation center given by the following expression:

(21) $$d_{\beta }\;=\;\frac{2\cdot \beta }{3}$$

Both β and σ_β_ are unknowns, so they must be determined in advance, in order to calculate *F*_β_ and *d*_β_ from Equations (20) and (21).

Assumption 2 implies that the concrete compressive strain ε_β_ at the internal circle (at *z* = *L* − *e*) is in proportion to the concrete compressive strain at the external circle (*z* = 0). The concrete compressive strain ε_β_ can thus be expressed as a function of λ and β:

(22) $$\varepsilon_{\beta }\;=\;0.0022\cdot \frac{\beta }{\lambda }$$

Assumptions 2 also entails that σ_β_ is elastic, which allows σ_β_ to be obtained by using the concrete elastic modulus *E_c_*:

(23) $$\sigma_{\beta }\;=\;0.0022\cdot \frac{\beta }{\lambda }\cdot E_{c}$$

where *E_c_* is provided by Equation (5).

Eventually, the force *F*_β_ can be expressed as:

(24) $$F_{\beta }\;=\;0.0011\cdot D\cdot \frac{\beta^{2}}{\lambda }\cdot E_{c}$$

The problem that has to be solved in order to determine the shear strength of an anchor group has two unknowns: 1- the position of the rotation center, whose distance is λ from the external circle and β from the internal circle of the circumscribing cylinder, where β + λ + *e* = *L* (which implies that the unknown is only one distance); 2- and the shear strength of the anchor group *V_gu_*, which is the prediction the model aims at.

The position of the rotation center can be obtained from the rotational equilibrium equation. Then, the shear strength *V_gu_* can be obtained from the translational equilibrium.

The rotational equilibrium equation is written with respect to the ends that protrude from concrete (free ends), so that the shear force, which now is unknown, is not included.

(25) $$F_{\beta }\cdot \left(d_{\beta }\;+\;\lambda \;+\;e\right)\;-\;F_{\lambda }\cdot \left(\lambda \;-\;d_{\lambda }\;+\;e\right)\;=\;0$$

Replacing Equations (14), (15), (20) and (21) into Equation (25), the following equation turns out.

(26) $$0.0011\cdot D\cdot \frac{\beta^{2}}{\lambda }\cdot E_{c}\cdot \left(\frac{2\cdot \beta }{3}\;+\;\lambda \;+\;e\right)\;-\;0.84\cdot {\left(\frac{f_{cm}}{33.0}\right)}^{0.11}\cdot D\cdot \lambda \cdot f_{cm}\cdot \left(0.42\cdot \lambda \;+\;e\right)\;=\;0$$

in which *f_cm_* is the biaxial compressive strength of concrete defined by Equation (19).

Equation (26) allows the diameter ϕ to be eliminated.

(27) $$0.0011\cdot \frac{\beta^{2}}{\lambda }\cdot E_{c}\cdot \left(\frac{2\cdot \beta }{3}\;+\;\lambda \;+\;e\right)\;-\;0.84\cdot {\left(\frac{f_{cm}}{33.0}\right)}^{0.11}\cdot \lambda \cdot f_{cm}\cdot \left(0.42\cdot \lambda \;+\;e\right)\;=\;0$$

Equation (27) shows that the position of the rotation center does not depend on the cylinder diameter, while it depends on the concrete constitutive law.

In Equation (24), *E_c_* and *f_cm_* are known, as they are provided by Equations (5) and (19), respectively, in which *f_c_* is data. Both β and λ are unknown, but the geometric parameters satisfy the relationship: β + λ + *e* = *L* (Figure 2), in which the length *e* belongs to the data. Thus, β can be expressed as a function of λ or vice versa. Ergo, Equation (27) includes only one unknown, which can be derived solving the equation. In so doing, β and λ are determined and, in turn, the position of the rotation center is determined.

The translational equilibrium equation that governs is that in the direction of the shear force:

(28) $$\begin{array}{l} F_{\beta }\;-\;F_{\lambda }\;+\;V_{ug}\;=\;0\;\;\;\;\;\to \\ V_{ug}\;=\;0.84\cdot {\left(\frac{f_{cm}}{33.0}\right)}^{0.11}\cdot \lambda \cdot \phi \cdot f_{cm}\;-\;0.0011\cdot \phi \cdot \frac{\;\beta^{2}}{\lambda }\cdot E_{c} \end{array}$$

The values of β and λ of Equation (28) are those obtained solving Equation (27). Once those values are put into Equation (28), the shear strength *V_gu_* of the anchor group embedded into concrete is gained and the problem is eventually solved.

Ultimately, the model—Equations (27) and (28)—requires knowing the uniaxial concrete strength *f_c_*, the length *L* and diameter ϕ of the anchor, and the length *e*, while *f_cm_* and *E_c_* are provided by Equations (5) and (19).

The substitution of β as a function of λ or vice versa in Equation (27), and the resulting explicit formula that gives λ or β is not presented here, since it would be not only cumbersome but above all useless. In fact, the Reader who will use this model will find easier to solve the two-equation system employing a mathematical program, which is what the author has performed in the case studies presented here.

## 7. Comparison between Model Results and Tests Results Borrowed from Literature

The predictive capacity of the analytical model stems from that of the non-linear numerical model, which was the tool used for performing a broad-spectrum analysis (Section 4), whose results have been reduced in five assumptions (Section 5) that reproduce the basic ultimate behaviors of an anchor group embedded into concrete. The analytical model has been derived from those assumptions, so that it is verified and validated on a theoretical basis.

In order to evaluate the predictive capacity of the analytical model not exclusively by theoretical means, experimental results were borrowed from literature and those tests were simulated by the analytical model of Section 6. The comparison between test and model results allowed the analytical model to be verified and validated on an experimental basis as well.

Unfortunately, most of the tests reported in the literature were performed on anchors cast into new structures, i.e., installed before the concrete is poured [16,68,69,77,86]. So, the anchors’ shear capacity of those tests was strengthened by nuts, washers, and/or plates attached to the embedded part. In addition, the concrete included specific bars and stirrups that embraced the anchors and strengthen their shear capacity too.

On the contrary, an anchor installed into hardened concrete consists of a straight shaft (shank), without any strengthening element. Moreover, the reinforcement of the member that the anchors are post-installed into was not designed to bear those anchors, which entails that the shear strength of the anchor group cannot rely on the reinforcement.

Thus, the tests included in this section are only those where the shear strength did not benefit of substantial extra-strength due to those devices.

The strength of the vast majority of the tested specimens reported in the literature was dictated by premature failure modes [20,21,22,23,24,25,26,27,28,29,30,31,32,33,34,35,36,37,38,39,40,41,42,43,44,45,46,47,48,49,50,51,52,53,54,55,56]. In fact, the mechanisms that occurred in most of the experimental tests were concrete pry-out failure, concrete splitting, concrete breakout, concrete edge failure, or anchor failure. So, the comparisons that could be set up in this research work could employ a minority of the experimental tests reported in the literature. Eventually, the tests included in this section are only those whose failure mode was pure shear mechanism with strength dictated by the concrete.

For the sake of completeness, it should also be mentioned that this research work included the comparison between the shear strength of some tests that collapsed by a premature failure mode, i.e., by edge effect [12,16], prying actions [41], or steel shear failure [9,61]. The theoretical shear strength predictions of the analytical model surpassed every homologous experimental shear strength. If those anchorages would have prevented those detrimental failure mechanisms from occurring, the shear strength would be expected to be close to that from the model.

In particular, the results of those comparisons agree with [12], whose main conclusion was that the shear strength of adhesive anchors in a multiple-anchor connection should be taken as 50% of the tensile strength and the shear strength of undercut anchors in a multiple-anchor connection should be taken as 60% of the tensile strength.

Reference [14] presented a series of tests of cast-in anchors subjected to simulated seismic loads, including 19 shear tests. Thirteen test blocks, with four anchors in each block, were prepared in that study. Namely, concrete block containing four anchor specimens were used in that research work, which implies that each specimen consisted of anchor groups with *N* = 4. The paper listed the characteristic dimensions for each anchor specimen and each block, so that sufficient information was provided to reproduce the experiments described.

The embedment depths (*L* − *e* = 102 mm and 152 mm) and edge distances of the anchor specimens were selected by the Authors targeting either steel fracture or concrete failure (shear strength based on the design capacity equations in ACI 318 [79]). As a result, 12 specimens of those 19 shear tests failed by pure shear mechanism dictated by the concrete. Therefore, those results could be used to verify this model.

The test anchor consisted of a 19 mm (3/4”) diameter ASTM F1554 Grade 55 threaded rod and a heavy hex nut at the embedded end, which both provided extra strength and extended the embedded length by adding 57 mm. The measured stress–strain relationship of the anchor’s steel indicated a yield strength of 476 N/mm^2^ and an ultimate strength of 527 N/mm^2^. Ready-mixed concrete with a specified compressive strength of 27.6 N/mm^2^ was designed. The actual compressive strength of the concrete was 46.1 N/mm^2^, measured using tests of three 100 × 200 mm cylinders with a coefficient of variation of 5.9% (the measures obtained from the cylinders were converted, so as to gain the actual compressive strength of the concrete).

The data of those 12 tests to plug into the analytical model are: *f_c_* = 46.1 N/mm^2^; *N* = 4; *L* = 169 mm and 219 mm; ϕ = 19 mm; *e* = 10.0 mm. The average shear strengths measured by the monotonic tests were *V_gu_* = 226.62 kN and 322.19 kN for the 169 mm and 219 mm length, respectively.

The shear strengths from the analytical model were *V_gu_* = 187.81 kN and 241.66 kN, respectively. The theoretical predictions resulted to be lower than the experimental results, as expected. The maximum difference between theoretical and experimental results turned out to be 17.1% and 25.0%, which is a satisfactory result considering the extra-strength given by the heavy hex nut, with respect to the anchor group simulated by the analytical model.

Load-controlled cyclic push-out tests were conducted in [48,93] as well. Stud shear fracture, due to low-cycle fatigue, was found to be the typical failure mode. The shear strengths from the analytical model resulted to be about 1.15 times as much as the homologous shear strength of the specimens that failed after considerable degradation of the load response (and increase in stud slips at failure were observed in the tests too).

Typically [14,15,32], the anchor shear capacity achieves about 80% of its monotonic capacity when the specimen is subjected to cyclic loads (in [14,15] degraded anchor capacity was also observed using push-out specimens and subjecting the anchors to displacement-controlled cyclic loading, and about the same differences were obtained). Ultimately, the analytical results agree satisfactory with those experimental results.

Reference [45] presented an experimental campaign which tested single ASTM A307 anchors under reversed cyclic shear loading. The tests with load-controlled loading confirmed that the anchor steel typically failed at much lower loads under cyclic loading than those tested monotonically. Again, shear strength of anchors under cyclic loadings resulted to be about 20% lower than that of anchors tested monotonically. The shear strengths from the analytical model resulted to be about 1.14 times as much as the homologous shear strength of the specimens tested cyclically, which is a satisfactory result in order to ascertain the predictive capacity and accuracy of the analytical model.

The comparisons were carried out on other experimental tests, which exhibited differences having the same order of magnitude of those presented above.

As a final remark about the comparison between analytical and experimental results, the latter validated the former, i.e., the analytical model presented in this paper satisfactory agrees with experiments borrowed from literature.

## 8. Practical Applications of the Model to Case Studies

This model was applied to a wide-ranging series of practical cases, in order to achieve two kinds of objectives. First, the case studies aimed to test the capacity of the analytical model of being easily applied. Second, the case studies aimed to explore the ultimate behavior of anchor groups embedded into concrete structures. The analysis of those results allowed a discussion to be developed and conclusions to be drawn (Section 9 and Section 10). Some of the case studies that were analyzed—data and results—are reported in Table 1.

As explained in the caption, each column of Table 1 describes a case study. According to Equations (27) and (28), hence, each column comprises the uniaxial concrete strength *f_c_* of the concrete that the anchors are embedded into, the length *L* of the anchors, the external protruding length *e*, the diameter of the circumscribing circle *D*—which are the data of the model—and the shear strength of the anchor group *V_gu_*—which is the result of the model. Table 1 is hence composed of five rows, plus the first row which reports the labels that identify each case study.

The cases of Table 1 were selected considering typical anchor groups, members, and concrete grades that are found in existing structures. Table 1 is devoted not only to presenting the results of some realistic and emblematic anchorages into concrete, but also to assisting the structural designer, since this table provides results that cover a wide variety of practical cases, which can help the structural engineer at the design stage.

The case studies of Table 1 were further analyzed, in order to point out the difference in strength of the anchors when they are part of a group (anchor group effect) with respect when they do not interact (no anchor group effect, i.e., *N* independent anchors). That analysis considered all the possible anchor patterns and diameters, and referred to the best combination of diameter and number of anchors that was found for each pattern. The results of that analysis are reported in Table 2.

As explained in the relevant caption, Table 2 associates a pattern to each anchor group of Table 1. The pattern includes the number *N* of anchors and their diameter ϕ (the anchors of each group are equal to each other). Then, the strength of each anchor group of Table 1 (i.e., the real strength, which is dictated by the group effect) was compared to the sum of the strengths of the *N* anchors of the group behaving as individual elements (no group effect).

The results of that analysis allowed the effect of the group on the anchors’ shear strength to be measured. To that end, the second to last row of Table 2 shows the shear strength of the anchor group *V_gu_* provided by this model (the output of Table 1), while the third to last row shows the product *N*·*V_u_*, which is denoted by Σ*^N^*·*V_u_*, where *V_u_* is the strength of the anchor as individual member (each anchor is assumed to be post-installed alone). The strength *V_u_* was calculated using a specific model of the author.

In order to quantify the penalization due to the anchor group effect, an exploitation ratio was introduced, which is defined as the ratio between *V_gu_* and Σ*^N^*·*V_u_*. That ratio is shown in the last row of Table 2.

## 9. Discussion

This section presents the critical discussion about the analytical model and about the major findings obtained from an analysis carried out using this model, which has included the case studies presented in Section 8 (Table 1 and Table 2) and other applications.

This research has studied anchor groups post-installed into concrete members, with adequate clearance from the edges, and subjected to transverse force. This paper has delivered an analytical model that simulates the ultimate behavior of an anchor group subjected to pure shear force.

The model presented in this paper consists of analytical exact mathematical formulation expressed by two closed-form equations, which allows the structural designer to predict the shear strength of an anchor group embedded into concrete. The model requires knowing one mechanical data, which is included in the basic information that must be available for sound analyzing an existing concrete building, and three geometric data related to anchor pattern and embedment.

More specifically, the model requires 4 pieces of data—namely, the uniaxial compressive strength of the concrete the anchors are embedded into, the diameter of the circumference that circumscribes the anchors, the embedded length of the anchors, and the length of the anchors that protrudes from the concrete surface connected to the attachment.

The analytical model presented here simulates anchors installed into hardened concrete, which entails that the anchor is a straight shaft (shank), with no hook at the embedded end, and with no nuts, washers, or plates attached to the embedded shaft. Differently from cast-in anchors, hence, post-installed anchors are not strengthened by those devices.

Moreover, the members that the anchors are post-installed into are existing, which entails that no bars and stirrups had been designed to bear the anchors. Differently from cast-in anchors, hence, post-installed anchors are not even strengthened by any reinforcement.

The analytical model derives from five mechanical assumptions. Consequently, the predictive capacity and accuracy of this model stems from the representativeness and accuracy of those assumptions. The mechanical assumptions were established based on a non-linear numerical analysis that simulated the behavior of a complete range of anchorages, which allowed the ultimate behavior of an anchor group to be described, comprehended, and interpreted. So, the assumptions are realistic and exhaustive. It follows that the model almost guarantees an equal degree of accuracy in predicting the shear strength of an anchor group.

Eventually, the numerical analysis allowed complexity to be accurately simulated, then to be reduced into five statements. In so doing, complexity was concentrated in the assumptions, so that the analytical model is easy and fast to use. Nevertheless, the accuracy of the model is that which is guaranteed by the assumptions.

That statement is confirmed by the comparisons to results of experimental tests found in literature (Section 7). It is hardly necessary to mention that the model was not calibrated against the experimental results, because it must describe the behavior of an any anchor group, and not only of the tested anchor groups. Namely, this is a predictive model. In other words, the experimental results borrowed from literature were used only to measure model accuracy and not to tune some parameters.

The final equations—Equations (27) and (28)—show that the shear strength of an anchor group depends on the diameter *D* of the circumscribing circumference, while it depends neither on the diameter of the anchors nor on the diameters of the individual drilled holes of each anchor. That conclusion holds true as long as the anchor group provides the circumscribing cylinder with substantial confinement, which can be guaranteed by the patterns of Figure 1 or equivalent patterns, together with close spacing or intermediate spacing (as defined in Section 4.1). Namely, the model can be used only for close or intermediate spacings, while for wide spacing the model to use is that for the individual anchor (in another research work, the author constructed its own analytical model for the individual anchors).

The results of the analytical model demonstrate that anchor group effect is different than what is usually referred to. Namely, the anchor group effect is not the phenomenon that causes the behavior an anchor group to be worse than the behavior that the anchors could display as individual components (i.e., without any group interaction). Actually, that behavior can be a consequence of anchor group effect, but is not the direct effect; hence, it is not its definition.

The direct effect of anchor group is to provide the concrete of the circumscribing cylinder with substantial confinement, which enhances its stiffness and strength.

Stiffness enhancement makes the circumscribing cylinder move as a rigid body, so that it rotates rigidly (while the rigid translation is marginal).

Strength enhancement makes the concrete of the circumscribing cylinder become much stronger than the concrete that surrounds the circumscribing cylinder, so that the shear strength is dictated by the latter but not by the former.

What reported above is the actual definition of anchor group effect that stems from this research work, while the consequence of this effect on the shear strength depends not only on anchor group effect (as defined here), but also on how much the anchors exploit the area of the circumscribing circumference.

When the area of the circumscribing circumference is highly exploited, the shear strength of the anchor group is lower than the sum of the shear strength of the anchors of the group as individual elements, i.e., without any group interaction. However, when the area of the circumscribing circumference is no more than moderately exploited, the shear strength of the anchor group is greater than the sum of the shear strengths of the anchors of the group as individual elements.

In Section 8, that behavior has been quantified by introducing a ratio that measures the level of exploitation of the circumscribing circumference. The numerator of that ratio is the anchor group shear strength, and the denominator is the sum of the shear strengths that each anchor would reach if it did not interact with the other anchors.

In the case studies of Table 2, that ratio resulted to be substantially lower than unit, as it can be as low as 0.60, and even lower (other cases analyzed not shown in the table). In those cases, hence, the anchor group effect significantly impacts on the shear strength and makes it substantially lower.

The case studies of Table 2 belong however to the category of close spaced anchors. Analyses carried out on intermediate spaced anchors demonstrated conversely that the ratio non only can be greater than unity but often is greater than unit.

The former result was expected, since it agrees with the conventional definition of anchor group effect, whereas the latter result was unexpected and is one of the novelties provided by this research work.

More specifically, this research work has proven that the anchor group effect consists of confining the concrete of the circumscribing cylinder, whereas its impact on the shear strength depends on the spacing of the anchors and can result either in a decrease or in an increase.

Those results allow the optimal design of an anchor group to be defined. Actually, there are two design approaches, according to the level of conservativeness that the designer wants to guarantee, i.e., according to the margin of safety that must be achieved.

For a drastically conservative safety assessment, the shear strength of the anchor group can be taken equal to the lowest value between the anchor group shear strength, i.e., *V_gu_*, and the sum of the shear strengths that each anchor would reach if it did not interact with the other anchors, i.e., Σ*^N^*·*V_u_* = *N*·*V_u_*. Under this assumption, an exploitation ratio greater than unity is useless.

For a realistic but safe safety assessment, the shear strength of the anchor group with either close spacing or intermediate spacings shall be taken equal to *V_gu_*, while for wide spacing the shear strength must be taken as Σ*^N^*·*V_u_* = *N*·*V_u_*. Under this assumption, an exploitation ratio greater than unity can be useful.

The former approach (the drastically conservative safety assessment) implies a specific design strategy for anchor groups. The circumscribing circumference is the starting point of the design, since it stems from issues that are at a higher level of the anchor design process and that being so it cannot be modified. Given the circumscribing circumference, the pattern and spacing of the anchor group shall be chosen so that the ratio is as close as possible to unity. In so doing, the number *N* and diameter ϕ are optimized. If, on the contrary, that ratio is lower than unity, the number and/or diameter of the anchors is/are excessive and can be reduced. While if the ratio is greater than unity, the area of the circumscribing circumference is not exploited, so its diameter *D* could be smaller.

The former approach implies hence finding the greatest exploitation ratio for the given circumscribing circumference.

The latter approach should start placing a minimum number of anchors and increasing the number up to reaching the strength that the anchor group has to transmit.

In the case studies of Table 2, the maximum shear stresses in the shaft of the anchors range from 50.0 N/mm^2^ to 100.0 N/mm^2^. The ultimate shear stress of all the anchors that can be found on the market is drastically greater than those shear stresses. Therefore, the shear strength of the case studies of Table 1 and Table 2 is dictated by the concrete that surrounds the circumscribing cylinder and not by the anchors (i.e., failure occurs by the crushing of the surrounding concrete and never by the rupture of the steel).

Model predictions were compared to the strength obtained from code provisions, requirements, specifications, standards. The comparisons revealed that the actual shear strength of an anchor group post-installed into concrete is substantially different than the shear strength furnished by documents edited by organizations for technical assessment of anchorages. The differences resulted to be even greater comparing model predictions to the strength from technical reports edited by producers and manufacturers.

Those differences are due to two facts. The first is that the formulations included into those documents are empirical, while this model is analytical, so it is more accurate. The second is that the tests from which the formulations were derived are devoted to anchorages for new structures (cast-in anchors), while this model simulates anchors whose strength cannot take advantage of any of the strengthening devices that are customarily used for anchors cast in new structures (the model simulates post-installed anchors).

One of the most popular approaches to the design of anchors embedded into concrete is Appendix B—Anchoring to concrete included in [80]. This appendix furnishes empirical formulas that predict the shear strength of an anchor group embedded into concrete. It is to not that [80] uses imperial units. Using the provisions of [80], the shear strength of each case studies reported in Table 1 and Table 2 was calculated so as to point out the differences. Unfortunately, [80] assumes that an anchor can fail due to the effects of proximity to edges, small depth of the concrete member, close spacing of anchors, or shear failure of the steel, whereas the model presented here considers anchors whose shear failure is not dictated by any premature failure mode. In order to minimize the conditions that lead to a premature failure mode, those code provisions were applied using a minimum distance from the edge of 200 mm (7.847 in), considering a shear force parallel to an edge, and assuming that the concrete is uncracked. Namely, code provisions were used providing the premature failure modes with the maximum strength that was possible.

The outputs of that analysis are presented in Table 3, which shows the comparisons between the results obtained using code provisions [80] and those obtained from this model. According to Table 3, the shear strengths from [80] are substantially different than those obtained from the analytical model presented here.

For large-sized anchors and strong concrete, the predictions of equations in ACI 349 [80] resulted to largely overestimate those of the analytical model. One of the reasons for those differences is that, as previously observed, the formulas of [80] are empirical and are devoted to anchors that include nuts, washers, plates, and special bars and stirrups that strengthen the shear capacity of the anchor.

On the contrary, for small size anchors and weak concrete, the predictions of equations in ACI 349 resulted to largely underestimate those of the analytical model. One of the reasons for those differences is that, when the concrete is weak, the edge effect and anchor group effect are substantial.

Since the 1990s, numerical structural analyses have almost replaced analytical methods in the profession. In particular, practitioners usually carry out structural analyses employing commercial software codes that apply the finite element methods, which also perform the final safety verifications according to a selected code.

This research work has proven that commercial software codes cannot correctly describe the ultimate behavior and reliably predict the shear strength of an anchor group embedded into concrete, and that they are not suited to use for assessing the safety conditions of an anchor group. Actually, to be accurate, a numerical model should be three-dimensional, and should use non-linear constitutive laws up to failure for concrete and interfaces, which goes beyond the capability of structural software systems directed at structural practice and used by practitioners.

The sole alternative to such numerical modeling is an analytical model derived from assumptions that accurately and exhaustively represent the ultimate behavior of anchor groups obtained from the numerical modeling. That is exactly what has been performed in this paper.

## 10. Conclusions

Activity included the verification that the model reproduces experiments present in the published literature with close agreement. The average difference between model simulations of experiments performed on cast-in anchors and the results of such experiments turned out to be approximatively 20%, which is a satisfactory result considering that the tested anchors benefitted from the extra-strength provided by nuts and washers attached to the embedded shaft.

Model predictions were also compared to code provisions and the differences turned out to be huge. In particular for large diameters model predictions resulted to be up to one third of the shear strengths calculated using code provisions, which proves that codes edited by organizations for technical assessment, as well as reports and instructions edited by producers and manufactures, are not reliable.

Accuracy of the model predictions depends on how much the analyzed anchor group is represented by the reference structure, which is an anchor groups that fails by pure shear strength of the surrounding concrete, whose anchors transmit no more than marginal axial force. Accordingly, strength assessment has also to ascertain that the bending moment transmitted by the anchor group from the attachment to the concrete is no more than moderate, so that the axial forces in the anchors be negligible. Moreover, the strength assessment has to ascertain that the edges do not imply any reduction in the strength (edge effects). Strength assessment has also to ascertain that the ultimate shear forces dictated by the steel of the anchors (rupture of the shafts) and by the anchoring material (adhesive anchors) are greater.

The results have shown that for close-spaced anchors the shear strength of the anchor group is lower than the sum of the shear strength of each single anchor of the group as individual member, while for intermediate spaced anchors the shear strength of the anchor group may be even higher than the sum of the shear strength of each single anchor as individual members.

The initial remark of this paper would also be suitable to close the paper. On one hand, post-installed anchors are marginally included in the papers present in the published scientific literature and in the documents edited by organizations for technical assessment, which are mainly devoted to cast-in anchors. On the other hand, the behavior of post-installed anchors is substantially different than the behavior of anchors cast in new concrete structures, i.e., before concrete has been poured. This paper has been written aiming to fill that gap for the anchor groups.

## Figures and Tables

**Table 1:** Case studies: shear strength of 18 anchor groups post-installed into concrete. The first row indicates the number of the case Study (S1–S18). The rows from the second to the second to last report the data of each case study (i.e., concrete compressive strength and anchor group geometry). The last row reports the shear strength *V_gu_* of each anchor group. The first and second columns report the symbol of the mechanical and geometrical parameters, and the relevant units. Each column from the third to the last reports a case study completely—data and result.

		S1	S2	S3	S4	S5	S6	S7	S8	S9	S10	S11	S12	S13	S14	S15	S16	S17	S18
*f_c_*	N/mm^2^	15.0	15.0	15.0	15.0	15.0	15.0	20.0	20.0	20.0	20.0	20.0	20.0	25.0	25.0	25.0	25.0	25.0	25.0
*L*	mm	195.0	195.0	195.0	270.0	270.0	270.0	218.0	218.0	218.0	225.0	225.0	225.0	192.5	192.5	192.5	235.5	235.0	235.0
*D*	mm	65.5	190.0	230.0	65.5	190.0	230.0	140.0	220.0	250.0	140.0	220.0	250.0	100.0	125.0	160.0	100.0	125.0	160.0
*e*	mm	15.0	15.0	15.0	20.0	20.0	20.0	18.0	18.0	18.0	25.0	25.0	25.0	12.5.0	12.5	12.5	15.5	15.0	15.0
*V_gu_*	kN	68.69	199.26	241.21	95.78	277.83	336.32	217.83	342.31	388.99	209.16	328.68	373.50	180.55	225.68	288.87	220.39	275.49	352.63

**Table 2:** Anchor groups versus individual anchors. Exploitation ratios of the 18 case studies of Table 1. The first row recalls the case study of Table 1 (S1–S18). The rows from the second to the fifth exhibit the value of the mechanical and geometrical parameters of each case study, which are those reported in Table 1. The sixth row reports the diameter ϕ of the anchors of each group. The seventh row reports the shear strength *V_u_* of the single anchor post-installed alone (no group effect). The eight row reports the number of anchors of the group and the nineth row reports the anchor pattern with reference to Figure 1 (the first digit is the row, and the second digit is the column of Figure 1). The combination of ϕ and *N* is the best for that pattern. The tenth row reports the strength that the anchor group would have if the anchor group effect were zero (no interaction between the *N* anchors), which is denoted by Σ*^N^V_u_*. According to the definition: Σ*^N^V_u_* = *N*·*V_u_*. The second to last row recalls the results of Table 1, i.e., this row exhibits the shear strength of the anchor group *V_gu_*. The last row reports the exploitation ratio, which, for each column, is the result of the second to last element divided by the third to last element. The first and second columns report the symbol of the mechanical and geometrical parameters, and the relevant units. Each column from the third to the last reports a case study completely and shows the role played by the anchor group effect in the shear strength of the anchor group.

		S1	S2	S3	S4	S5	S6	S7	S8	S9	S10	S11	S12	S13	S14	S15	S16	S17	S18
*f_c_*	N/mm^2^	15.0	15.0	15.0	15.0	15.0	15.0	20.0	20.0	20.0	20.0	20.0	20.0	25.0	25.0	25.0	25.0	25.0	25.0
*L*	mm	195.0	195.0	195.0	270.0	270.0	270.0	218.0	218.0	218.0	225.0	225.0	225.0	192.5	192.5	192.5	235.5	235.5	235.5
*D*	mm	65.5	190.0	230.0	65.5	190.0	230.0	140.0	220.0	250.0	140.0	220.0	250.0	100.0	125.0	160.0	100.0	125.0	160.0
*e*	mm	15.0	15.0	15.0	20.0	20.0	20.0	18.0	18.0	18.0	25.0	25.0	25.0	12.5	12.5	12.5	15.5	15.0	15.0
ϕ	mm	14.0	28.0	34.0	16.0	39.0	33.0	20.0	33.0	33.0	24.0	26.0	30.0	39.0	33.0	35.0	28.0	24.0	20.0
*V_u_*	kN	14.68	29.36	35.66	23.40	57.03	48.25	31.12	51.35	43.57	35.86	38.84	44.82	70.41	59.58	46.94	61.71	52.89	44.08
*N*	/	6	7	8	6	6	8	9	10	12	7	10	9	3	5	8	6	8	12
Pattern	Figure 1	3-1	3-2	3-3	2-3	3-1	3-3	4-1	4-2	4-3	3-2	4-2	4-1	1-1	2-2	3-3	2-3	3-3	4-3
Σ*^N^ V_u_*	kN	88.08	205.52	285.28	140.40	342.18	386.00	280.08	513.50	522.84	251.02	388.41	403.38	211.23	297.90	375.52	370.26	423.12	528.96
*V_gu_*	kN	68.69	199.26	241.21	95.78	277.83	336.32	217.83	342.31	388.99	209.16	328.68	373.50	180.55	225.68	288.87	220.39	275.49	352.63
Ratio	/	0.78	0.97	0.85	0.68	0.81	0.87	0.78	0.67	0.74	0.83	0.85	0.93	0.86	0.76	0.77	0.60	0.65	0.67

**Table 3:** Comparisons between results from this analytical model and results from code provisions. First row: number of the case Study (S1–S18), which are those of Table 1. Second row: *V_gu_* from the analytical model, i.e., shear strengths of the anchor groups reported in Table 1. Third row: shear strength from Appendix B of the Aci code [80], which is denoted by *Vgu-a*. Each column compares thus the analytical result to the Aci code provision for each case study of Table 1.

		S1	S2	S3	S4	S5	S6	S7	S8	S9	S10	S11	S12	S13	S14	S15	S16	S17	S18
*V_gu_*	kN	68.69	199.26	241.21	95.78	277.83	336.32	217.83	342.31	388.99	209.16	328.68	373.50	180.55	225.68	288.87	220.39	275.49	352.63
*V_gu-a_*	kN	44.34	117.56	399.20	61.03	544.22	851.60	283.18	600.55	1011.11	299.98	547.43	784.35	352.83	335.36	707.09	579.84	459.15	1100.12
